# A computational framework for modeling cell–matrix interactions in soft biological tissues

**DOI:** 10.1007/s10237-021-01480-2

**Published:** 2021-06-25

**Authors:** Jonas F. Eichinger, Maximilian J. Grill, Iman Davoodi Kermani, Roland C. Aydin, Wolfgang A. Wall, Jay D. Humphrey, Christian J. Cyron

**Affiliations:** 1 Institute for Computational Mechanics, Technical University of Munich, Garching, 85748 Germany; 2 Institute for Continuum and Materials Mechanics, Hamburg University of Technology, Hamburg, 21073 Germany; 3 Department of Biomedical Engineering, Yale University, New Haven, CT 06520 USA; 4 Institute of Material Systems Modeling, Helmholtz-Zentrum Hereon, Geesthacht, 21502 Germany

**Keywords:** mechanical homeostasis, growth and remodeling, cell–extracellular matrix interaction, discrete fiber model, finite element method

## Abstract

Living soft tissues appear to promote the development and maintenance of a preferred mechanical state within a defined tolerance around a so-called set point. This phenomenon is often referred to as mechanical homeostasis. In contradiction to the prominent role of mechanical homeostasis in various (patho)physiological processes, its underlying micromechanical mechanisms acting on the level of individual cells and fibers remain poorly understood, especially how these mechanisms on the microscale lead to what we macroscopically call mechanical homeostasis. Here, we present a novel computational framework based on the finite element method that is constructed bottom up, that is, it models key mechanobiological mechanisms such as actin cytoskeleton contraction and molecular clutch behavior of individual cells interacting with a reconstructed three-dimensional extracellular fiber matrix. The framework reproduces many experimental observations regarding mechanical homeostasis on short time scales (hours), in which the deposition and degradation of extracellular matrix can largely be neglected. This model can serve as a systematic tool for future *in silico* studies of the origin of the numerous still unexplained experimental observations about mechanical homeostasis.

## Introduction

Living soft tissues, in contrast to classical engineering materials, usually seek to establish and maintain a mechanical state that is not stress-free. This behavior of living soft tissues is often referred to as *mechanical homeostasis*, and it plays a key role in the control of form and function in health and disease (Lu et al. 2011; Cox and Erler 2011; Ross et al. 2013; Humphrey et al. 2014; Bonnans et al. 2014). Intracellular structures such as the actomyosin cytoskeleton are physically coupled to the surrounding extracellular matrix (ECM) via transmembrane protein complexes such as integrins that can cluster to form focal adhesions (Cavalcanti-Adam et al. 2007; Lerche et al. 2019). This coupling allows cells to receive mechanical cues from their environment, transduce these cues into intracellular signals, and react, for example, by adapting cellular stress and thereby also the stress of the surrounding ECM. Physical interactions between cells and ECM have been shown to control various processes on the cellular scale such as cell migration (Grinnell and Petroll 2010; Xie et al. 2017; Hall et al. 2016; Kim et al. 2020), differentiation (Chiquet et al. 2009; Mammoto et al. 2012; Zemel 2015; Seo et al. 2020), and survival (Bates et al. 1995; Schwartz 1995; Zhu et al. 2001; Sukharev and Sachs 2012) and are therefore fundamental for health and in disease of entire tissues and organs.

To study the micromechanical foundations of mechanical homeostasis experimentally, tissue culture studies with cell-seeded collagen or fibrin gels have attracted increasing interest over the past decades (Eichinger et al. 2021). Circular free-floating gels, when seeded with fibroblasts, exhibit a strong compaction over multiple days in culture due to cellular contractile forces (Simon et al. 2012, 2014). Studies of such gels whose compaction is prevented by boundary constraints typically show a two-phase response. First, tension in the gels rapidly increases to a specific value, the so-called homeostatic tension (phase I), and then remains largely constant (phase II) for the rest of the experiment (Brown et al. 1998, 2002; Sethi et al. 2002; Campbell et al. 2003; Marenzana et al. 2006; Dahlmann-Noor et al. 2007; Karamichos et al. 2007; Ezra et al. 2010; Courderot-Masuyer 2017; Eichinger et al. 2020). If the gel is perturbed in phase II, for example, by an externally imposed deformation, cells appear to promote a restoration of the homeostatic state (Brown et al. 1998; Ezra et al. 2010). Despite substantial research efforts over decades, the exact interplay between cells and surrounding tissue that is crucial for mechanical homeostasis and other related phenomena such as durotaxis still remains poorly understood (Eichinger et al. 2021).

Computational studies in this field have focused primarily on decellularized ECM systems to study the micromechanical and physical properties of networks of fibers (Heussinger and Frey 2007; Mickel et al. 2008; Lindström et al. 2010; Chatterjee 2010; Broedersz et al. 2011; Stein et al. 2010; Cyron and Wall 2012; Cyron et al. 2013b, a; Lang et al. 2013; Motte and Kaufman 2013; Müller et al. 2014; Jones et al. 2014; Lee et al. 2014; Müller et al. 2015; Ronceray et al. 2016; Mauri et al. 2016; Dong et al. 2017; Humphries et al. 2018; Zhou et al. 2018; Bircher et al. 2019; Domaschke et al. 2019, 2020). Current computational models of cell–ECM interactions often suffer from shortcomings—most are limited to two dimensions and just one or two cells (Wang et al. 2014; Abhilash et al. 2014; Notbohm et al. 2015; Jones et al. 2015; Kim et al. 2017; Humphries et al. 2017; Grimmer and Notbohm 2017; Burkel et al. 2018). The importance of the third dimension for the physics of fiber networks is well known (Cukierman et al. 2002; Baker and Chen 2012; Jansen et al. 2015; Duval et al. 2017), and it can be assumed that collective interactions between more than just two cells play important roles in mechanical homeostasis. Moreover, current models typically rely in many crucial aspects on heuristic assumptions (Nan et al. 2018; Zheng et al. 2019) and almost all of them assume simple random fiber networks (e.g., based on Voronoi tessellations) that do not match the specific microstructural characteristics of actual collagen gels or tissues. What remains wanting is a robust, computationally efficient three-dimensional model of cell–fiber interactions, where the microstructure of the fiber network realistically resembles real collagen gels and tissues and which is efficient enough to enable simulations with several cells. Such a computational model can be expected to help unravel the micromechanical and molecular foundations of mechanical homeostasis.

In this paper, we introduce such a computational model. It is based on the finite element method and relies on a strong experimental foundation. It can be used to test various hypotheses with regard to the micromechanical principles of mechanical homeostasis. It can also help to identify promising future experiments. The model focuses on mechanical aspects of homeostasis by concentrating on the physical interactions of cells with surrounding matrix fibers and thus neglects direct modeling of biochemical phenomena. The paper focuses on a detailed description of the computational framework, but examples are used to demonstrate the physical validity of this framework and to illustrate the opportunities it will open up. It will be seen that this framework captures well key observations from experiments on short time scales (in which deposition and degradation of tissue fibers can be neglected), thus helping to explain the underlying physics.

## Models and methods

To study the physical foundations of mechanical homeostasis in soft biological tissues on short time scales (hours), our framework models (i) interlinked ECM-like fiber networks whose microstructure closely resembles that of actual collagen gels, (ii) transmembrane proteins such as integrins which connect extra- to intracellular structures, and (iii) the contractile activity of the cytoskeleton. In the following, we describe the mathematical and computational details of our model.

### Construction of representative volume elements (RVEs)

**Fig. 1:**
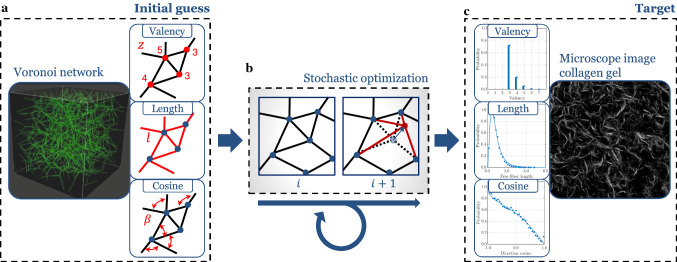
Schematic of the network construction process. **a** Random fiber network geometries based on Voronoi tessellation are used as the initial configuration. Valency, length, and cosine distribution are used as descriptors of the network geometry for which target distributions are given. **b** By iterative random displacements of arbitrary nodes in the network and accepting these displacements based on their impact on the system energy, which penalizes deviations of the geometric descriptors from their target distributions, one arrives after a number of stochastic steps at a configuration with the desired distribution of the geometric descriptors of interest. **c** Microscope images of collagen gels are used to determine the target distributions for the descriptors of the network

Computational modeling of soft tissues on the level of discrete fibers and individual cells remains intractable for large tissue volumes, noting that $1\, \rm{ml}$ of ECM may contain over one million cells. Therefore, we use RVEs as structurally typical samples of the considered tissue (Fig. 1a). Building on our previous work on biopolymer networks (Cyron and Wall 2012; Cyron et al. 2013a, b; Grill et al. 2021), we constructed physically realistic three-dimensional fiber networks from confocal microscope images of actual collagen gels (Fig. 1a). Following Lindström et al. (2010) and Davoodi-Kermani et al. (2021), we assumed that the mechanical properties of collagen fiber networks are predominantly governed by three descriptors, namely the valency (number of fibers connected to a network node, referred to by some as *connectivity*), the free-fiber lengths between adjacent nodes (herein also referred to as fiber length), and the angles between the fibers joining at the nodes (which can be quantified by the cosine of the angles between any pair of fibers joining at a node). These descriptors vary in the network across the fibers and nodes by following certain statistical distributions. Using the computational procedure described in Appendix A1, which is motivated by Yeong and Torquato (1998) and Lindström et al. (2010), and briefly illustrated in Fig. 1, we ensured that the statistical distributions of valency, free-fiber length, and inter-fiber cosines closely matched those of actual collagen fiber networks. The computational procedure to produce such networks has been implemented in a short C++ program which is available under the BSD 3-Clause License as the repository BioNetGen hosted at https://github.com/bionetgen/bionetgen.

### Mechanical network model

We used the finite element method to model the mechanics of our fibrous RVEs. Individual fibers were modeled as geometrically exact beam finite elements based on the nonlinear Simo–Reissner theory (Reissner 1981; Simo 1985; Simo and Vu-Quoc 1986) and a hyperelastic material law. This beam theory captures the modes of axial tension, torsion, bending, and shear deformation and is appropriate for large deformations. Thus, our finite element model of the fiber network can capture all essential modes of mechanical deformation. If not stated otherwise, covalent bonds between fibers were modeled as rigid joints coupling both translations and rotations. We chose the dimensional and constitutive parameters to mimic collagen type I fibers as the most abundant structural protein of the ECM. Fibers are assumed to have circular cross sections with a diameter of $D_f = 180\ \rm{nm}$ (Van Der Rijt et al. 2006) and elastic moduli of $E_f = 1.1\ \rm{MPa}$ (Jansen et al. 2018). Assuming curvilinear fibers with circular cross section of diameter $D_f$, the average mass density of collagen $\rho _{c}$ in the network RVE was calculated as

1 $$\begin{aligned} \rho _{c}&= \frac{L_{tot}{D_f}^{2} \pi }{V_{\text {RVE}} v_{c}} \end{aligned}$$

according to Stein et al. (2008), where $L_{tot}$ is the sum of all individual fiber lengths, $V_{\text {RVE}}$ the volume of the RVE, and $v_{c} = 0.73 \ \text {ml/g}$ the specific volume of collagen fibers (Hulmes 1979).

### Fiber to fiber cross-linking

A native ECM consists of myriad structural constituents, including collagen and elastin, which usually form networks to provide mechanical support to the resident cells. To form these networks, covalent cross-links are formed via the action of enzymes such as lysyl oxidase and transglutaminase, which can be produced by the cells (Simon et al. 2014). In addition to covalent bonds, transient hydrogen bonds or van der Waals bonds contribute further to the mechanical integrity of the ECM (Kim et al. 2017; Ban et al. 2018).

**Fig. 2:**
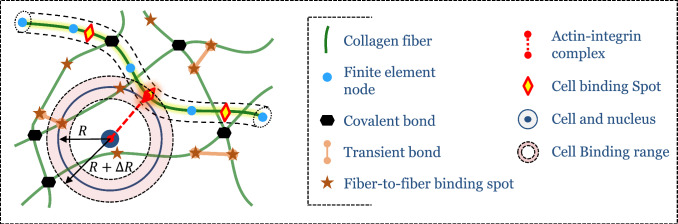
Fiber network model: collagen fibers are modeled as beam-like mechanical continua discretized by beam finite elements. Nearby collagen fibers are connected by permanent (covalent) chemical bonds modeled as rigid joints. During the simulation, additional transient bonds may stochastically form and dissolve between nearby binding partners on the fibers. These bonds are also modeled by short beam elements transmitting forces and moments. Cells of radius *R* can attach to nearby collagen fibers if certain predefined cell binding locations on the surrounding fibers are within $R-\Delta R$ and $R+\Delta R$ around the cell

To model initially existing covalent bonds between fibers, we permanently connect individual fibers joining at nodes of our initially generated network by rigid joints. To model the formation of additional transient and covalent bonds, we define so-called binding spots on all fibers (Fig. 2). If during the simulation it happens that the distance between two binding spots on distinct filaments falls within a certain critical interval, a new bond between the two filaments is established according to a Poisson process with an on-rate $k_{on}^{f-f}$. That is, within a subsequent time step $\Delta t$, a bond is assumed to form with the probability

2 $$\begin{aligned} p_{on}^{f-f}&=1 - \exp {(-k_{on}^{f-f}\Delta t)}. \end{aligned}$$

Newly established bonds are modeled by initially stress-free beam elements. Bonds established this way during the simulation can also dissolve. This process is again modeled by a Poisson process with an off-rate $k_{off}^{f-f}$, yielding in each time step $\Delta t$ an unbinding probability

3 $$\begin{aligned} p_{off}^{f-f}(F)&=1 - \exp {(-k_{off}^{f-f}(F)\Delta t)}. \end{aligned}$$

The off-rate is in general affected by the force *F* acting on the bond because transient chemical bonds under mechanical loading are typically less (though in certain regimes more) stable than load-free bonds (Bell 1980). This phenomenon can be modeled by a force-dependent off-rate

4 $$\begin{aligned} k_{off}^{f-f}(F)&= k_{off,0}^{f-f}\ \exp {\left( \frac{F \Delta x}{k_B T}\right) }, \end{aligned}$$

with $\Delta x$ a characteristic distance, $k_B$ the Boltzmann constant, and *T* the absolute temperature (Bell 1980). $\Delta x > 0$ was chosen so that the bond weakens under tension, a bond behavior that is often referred to as slip-bond behavior. By choosing $k^{f-f}_{off,0} = 0$, we can model new covalent bonds formed during our simulations, whereas $k^{f-f}_{off,0} > 0$ mimics transient bonds.

### Cell–ECM interaction

**Fig. 3:**
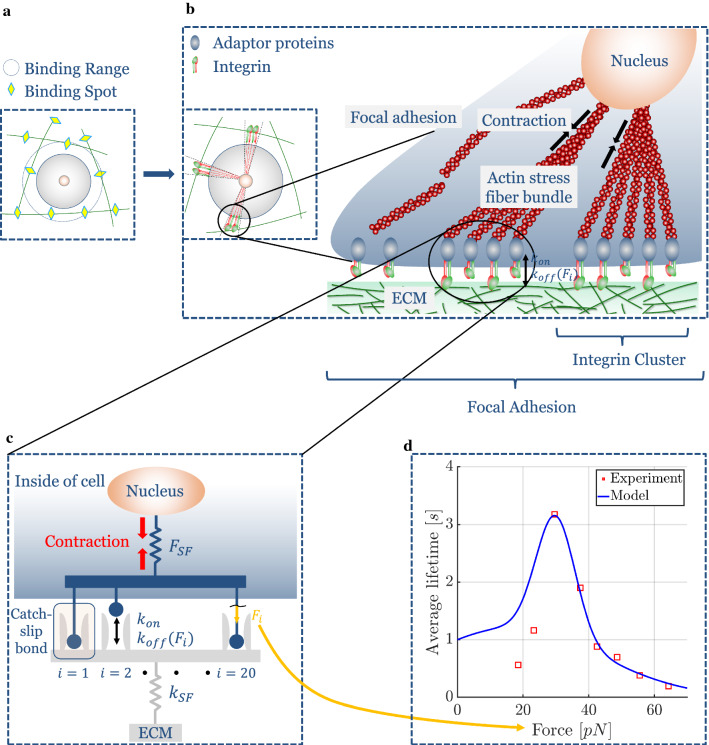
**a** If cells lie within a certain distance from integrin binding spots on fibers, a focal adhesion can from with a certain probability. **b** A focal adhesion consists of around 1000 integrins connecting the intracellular actin cytoskeleton to the ECM fibers. Actin stress fibers connect the cell nucleus to the focal adhesions and are modeled as elastic springs that contract over time. **c** Each focal adhesion consists of numerous so-called integrin clusters, each formed by $20-50$ integrins. We assume that each integrin cluster is connected to one actin stress fiber. Integrins are modeled as molecular clutches, i.e., they bind and unbind according to specific binding kinetics. **d** Experiments have determined a catch–slip bond behavior for single integrins where the lifetime does not monotonically decrease with the mechanical force transmitted through the bonds but where there exists a regime where increasing forces increase the average lifetime of the bond. To avoid infinite off-rates in case of low forces, we chose a slightly higher lifetime for low forces compared to the experimental data of Kong et al. (2009)

Cells in soft tissues can mechanically connect to surrounding fibers by integrins and exert stress on them via focal adhesions. A focal adhesion usually includes an actin stress fiber bundle in the cytoskeleton that connects the nucleus of the cell with the integrins of a cluster and can actively contract. Based on experimental observations, we restricted the maximal number of focal adhesions per cell to $N_{FA,max}=65$ (Kim and Wirtz 2013; Horzum et al. 2014; Mason et al. 2019). Figure 3b on the left shows three focal adhesions. It has been shown experimentally that roughly $N_{i,FA,max} = 1000$ integrins are involved in one focal adhesion (Wiseman 2004; Elosegui-Artola et al. 2014). These integrins are organized in so-called integrin clusters of roughly $20-50$ integrins (Changede et al. 2015; Cheng et al. 2020) (Fig. 3c). We thus assume for each focal adhesion 50 integrin clusters containing a maximum of $N_{i,ic,max} = 20$ integrins each.

To model cell-mediated active mechanical processes in soft tissues, we model the cell centers as point-like particles. When these particles approach predefined integrin binding partners (with a distance of $$d^{i-f} = 50$$ nm to each other; López-García et al. 2010) on the fibers within $$\pm \Delta R$$ around the cell radius *R*, a physical connection between cells and fibers is assumed to form by a Poisson process similar to the one in Eq. (2), but with a specific on-rate $$k_{on}^{c-f}$$ (see also Fig. 3a). The actin stress fibers connecting the cell nucleus with the fibers surrounding the cells are modeled as elastic springs (Fig. 3b, c) whose stress-free length evolves at some predefined rate $${\dot{c}}$$ that can be calculated to match experimental data of different cell types. These stress fibers contract at a rate of $${\dot{c}} = 0.1\ \text{$\mu$m/s}$$ (Choquet et al. 1997; Moore et al. 2010). The force acting on a single integrin $$F_i$$ can be computed according to

5 $$\begin{aligned} F_i = \frac{F_{SF}}{N_{i,bonded}}, \end{aligned}$$

with $F_{SF}$ the force acting in the respective stress fiber and $N_{i,bonded}$ the number of currently bound integrins in the integrin cluster associated with the respective stress fiber.

In contrast to many previous approaches in which displacements have been prescribed in the neighborhood of cells to model their contraction, we are able to model a true two-way feedback loop between cell and ECM. Integrins have been shown experimentally to exhibit a so-called catch–slip bond behavior (Kong et al. 2009) whose unbinding can be modeled by a Poisson process with a force-dependent off-rate

6 $$\begin{aligned} \begin{aligned} k_{off}^{c-f}(F) = a_1\text {exp}\left( -\left( \frac{F-b_1}{c_1}\right) ^2\right) \\ + a_2\text {exp}\left( -\left( \frac{F-b_2}{c_2}\right) ^2\right) \end{aligned} \end{aligned}$$

whose parameters were determined via fits to the experimental data (Kong et al. 2009; Weng et al. 2016) as shown in Fig. 3d and in Table 2. While the average lifetime of most chemical bonds decreases monotonically with increasing force transmitted by the bond, catch-slip bonds exhibit a regime where the bond stabilizes as the force increases. As illustrated in Fig. 3d, this makes integrin bonds particularly stable for values of $F_i$ in a range around 30 pN. Recall that we model an integrin cluster as a system of 20 parallel integrins whose bonds form and dissolve according to the above specified on- and off-rates (Fig. 3c). If at a certain point all bonds happen to have broken at the same time, the related integrin cluster is assumed to dissolve. It may, however, reform on the basis of a new (not yet contracted) stress fiber shortly thereafter with a binding rate $k_{on}^{c-f}$. If all clusters of a certain focal adhesion happen to dissolve at the same time, the focal adhesion as a whole is dissolved.

This model implies that many binding and unbinding events of integrins occur during the lifetime of a focal adhesion. This way, our model captures the chemical dynamics of the connection between cells and ECM fibers on different scales ranging from individual integrins to whole focal adhesions (Stehbens and Wittmann 2014). Thereby, our model both captures typical lifetimes of focal adhesions on the order of minutes and turnover rates of most proteins involved in the adhesion complex on the order of seconds.

### Boundary conditions

As mentioned before, simulations of complete tissues on the *cm*-scale are computationally expensive with discrete fiber models; hence, we study RVEs. A major challenge in the context of discrete fiber simulations is the imposition of deformations on the RVE to study its response to certain strains. To this end, most previous work by others requires that the nodes of the finite elements used to discretize fibers are located exactly on the boundary surfaces of the RVE where displacements are prescribed (Stein et al. 2010; Abhilash et al. 2014; Liang et al. 2016; Humphries et al. 2018; Burkel et al. 2018; Ban et al. 2018, 2019). Other approaches prescribe the displacements of nodes close to these surfaces (Lee et al. 2014). These methods share the problem that they do not ensure full periodicity across the boundaries where displacements are prescribed. To overcome this limitation, we developed a novel form of fully periodic boundary conditions for fiber networks that allows imposition of complex multiaxial loading states. This approach ensures full periodicity across all surfaces of the RVE and thereby minimizes computational artifacts due to finite-volume effects. The computational details of our algorithm are summarized in Appendix A1. Briefly, every point on a fiber that would reside outside of the RVE in the *i*-th coordinate direction is shifted back in by the length of the RVE in the respective direction $L_i$ (Fig. 11a). In this way, Dirichlet boundary conditions can be applied by simply stretching the RVE as a whole, as this results in a strain in each fiber that is cut by the boundary in the direction of the applied load due to a change in the shifting factor (Fig. 11c, d).

### Search algorithm and parallel computing

To yield meaningful computational results, the RVEs have to be much larger than the characteristic microstructural features such as the free-fiber length between adjacent nodes. Using values for the cell density and collagen concentration in a physiologically reasonable range typically leads to a system size of the RVE that can be solved only by parallel computing, including an efficient parallel search algorithm for the evaluation of all interactions between cells and fibers. We implemented such a search algorithm based on a geometrical decomposition of the computational domain in uniform cubic subdomains. The computational details of our parallelization are summarized in Appendix A2. Importantly, our approach does not require any fully redundant information on all processes, which enables a highly efficient parallelization on even a very large number of processors.

## Results and discussion

The presented computational framework was implemented in our in-house research finite element code BACI 2021. To ensure robustness, scalability, and especially validity, we performed various computational simulations and compared the results with available experimental data. The default parameters used in our simulations are listed in Table 2.

### Network construction

**Fig. 4:**
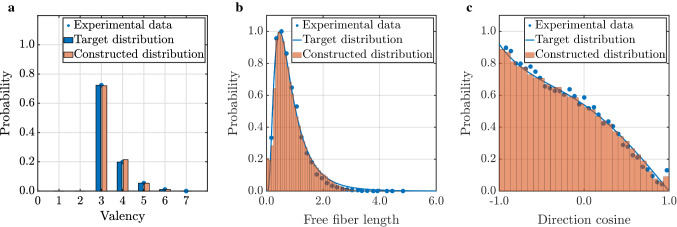
Results of the network construction process for a collagen concentration of $2.5$ mg/ml. **a** valency distribution, **b** free-fiber length distribution and **c** cosine distribution fit well the target distributions defined on the basis of experimental data taken from Nan et al. (2018) in **a** and from Lindström et al. (2010) in **b** and **c**

**Fig. 5:**
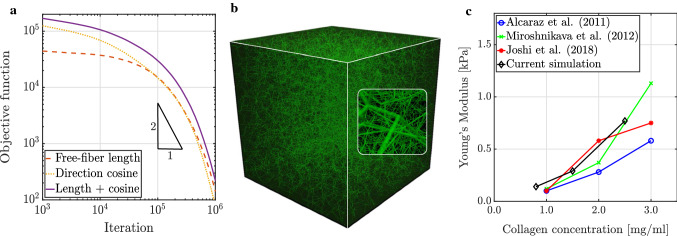
**a** In the stochastic network construction with a collagen concentration of $$0.8$$ mg/ml in a cube of edge length $$245\ \text{$\mu$m}$$, the energy-type objective function according to Eq. (11) is reduced during simulated annealing (in the studied range even superquadratically) by multiple orders of magnitude; **b** this optimization process yields RVEs with a desired microstructure; **c** the effective Young’s moduli at strains $$< 1\%$$ of RVEs constructed this way match well with the ones observed in experiments (Alcaraz et al. 2011; Miroshnikova et al. 2011; Joshi et al. 2018)

We first validated the network generation method described in Sect. 2. To this end, we created networks with different collagen concentrations and target descriptor distributions as observed by confocal microscopy in tissue culture experiments with collagen type I gels (Lindström et al. 2010; Nan et al. 2018). As shown in Fig. 4, our stochastic optimization method successfully generates networks with the desired distributions of valency, free-fiber length, and cosine. Figure 5a demonstrates that our simulated annealing converged well toward the desired network (Fig. 5b) with an increasing number of random iteration steps.

### Passive mechanical properties: stiffness

Next, we verified that our constructed, still acellular, networks have similar mechanical properties as actual collagen networks. To this end, we simulated simple uniaxial tensile tests with different collagen concentrations and compared the resulting values for the stiffness with values that have been collected in uniaxial experiments with collagen type I gels (Alcaraz et al. 2011; Miroshnikova et al. 2011; Joshi et al. 2018). We stretched a cubic simulation box with edge length $$L = 245\ \text{$\mu$m}$$ in one direction by applying displacement boundary conditions as described in Appendix A1 at a slow loading rate of $$0.01\ \text{$\mu$m/s}$$ up to a strain of $$1.0 \%$$; strains around $$1 \%$$ have been shown to be the relevant range when studying active, cell-mediated force development (Eichinger et al. 2020). Figure 5c demonstrates that the Young’s moduli of the constructed networks match well with the values observed in tissue culture experiments. In our artificial RVEs, we found a power law dependence between the Young’s modulus and the collagen concentration with an exponent of 1.33, similar to the exponent of 1.22 found experimentally (Joshi et al. 2018).

### Active mechanical properties: homeostatic tension

In this section, we consider cell-seeded fiber networks to study the *active* mechanics of soft tissues. The tension that develops in constrained gels stems from the contractile forces exerted by the cells on the surrounding fibers. In initially stress-free collagen gels seeded with fibroblasts, the tension builds up over a few hours until it has reached a plateau value, the so-called homeostatic value (Brown et al. 1998, 2002; Sethi et al. 2002; Campbell et al. 2003; Marenzana et al. 2006; Dahlmann-Noor et al. 2007; Karamichos et al. 2007; Ezra et al. 2010; Courderot-Masuyer 2017; Eichinger et al. 2020). Tissue culture experiments (Delvoye et al. 1991; Eichinger et al. 2020) have shown that the homeostatic tension depends on both cell and collagen concentration in the gel. We used this observation to validate our computational model. We created RVEs with an edge length of $$L = 245\ \text{$\mu$m}$$ and three different cell densities and collagen concentrations as studied experimentally in Eichinger et al. (2020). To increase the complexity of the RVE only gradually by adding cells, we still solely considered covalent bonds between matrix fibers. We then compared the cell-mediated active tension over time of our simulations to the one observed experimentally.

It is important to note that a direct (quantitative) comparison between experimental data and simulation results is difficult due to differing boundary conditions. Tissue culture experiments have at least one traction-free boundary (uniaxial gels have two, circular discs three), while we performed our simulations with RVEs with periodic boundary conditions applied in all directions. Note also that a free boundary in a microscopic RVE would not resemble a free boundary of a macroscopic specimen. It has been shown, however, that the number of fixed boundaries has a crucial impact on the homeostatic plateau value (Eichinger et al. 2020). In the following, we compare the first Piola–Kirchhoff stresses as the thickness of the gel samples over time is unknown. An initial thickness of the gel of $$t_{initial} = $$ 1.6 mm (knowing it to be between 1.0 mm and 3.0 mm) is assumed to fit best to the simulation data presented in the following. The initial width of the undeformed gel is 10 mm. The stresses for the RVEs were quantified as the sum of all fiber tractions across a boundary divided by the respective cross-sectional area.

#### Variation of cell density

**Fig. 6:**
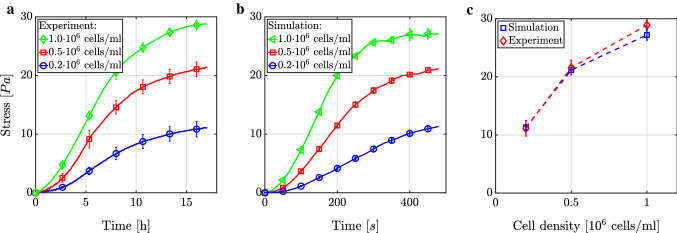
For a collagen concentration of $1.5\ \text {mg/ml}$, we compare the development of first Piola–Kirchhoff stress in **a** experiments (Eichinger et al. 2020) and **b** simulations. A good semiquantitative agreement of the expected cell-mediated steady state with nonzero tension (last data points of **a** and **b**) is observed **c**, however, also a significant difference of the time scales. All lines show the mean ± standard error of the mean (SEM) of three identical experiments in **a** and **c** and of three simulations with different random network geometries in **b** and **c**

In this section, we consider gels with a constant collagen density of 1.5 mg/ml. Cell densities of $0.2 \cdot 10^6\ \text {cells/ml}$, $0.5 \cdot 10^6\ \text {cells/ml}$ , and 1.0 $\cdot 10^6\ \text {cells/ml}$ studied in Eichinger et al. (2020) translate in our simulations into 3, 8, and 15 cells per RVE, respectively. Figure 6a shows the evolution of first Piola–Kirchhoff stress (true force/original area) generated in uniaxially constrained, dog-bone-shaped collagen gels as observed experimentally. The gradient during the first 10 h of the experiment and the homeostatic plateau level of stress increase with cell density. Both features are observed in our simulations and fit quantitatively well (Fig. 6b, c). We can therefore conclude that actin cytoskeleton contraction and the focal adhesion dynamics described in Sect. 2.4 are sufficient mechanisms to reproduce this non-trivial relationship.

A crucial difference between experiments and simulations is the time scale. Whereas mechanical homeostasis develops over a couple of hours in the experiments, it does so within a couple of minutes in the simulation. Interestingly, this time scale of our simulations agrees well with that for which single cells in experiments on purely elastic substrates reach a homeostatic state (Weng et al. 2016; Hippler et al. 2020). Thus, a possible explanation for the difference between our simulations and the experimental data from Eichinger et al. (2020) may be that in tissues with numerous cells, complex interactions between the cells substantially delay the homeostatic state. Such interactions remain poorly understood and are not yet accounted for in our computational framework. Another possible explanation for the different time scales in Fig. 6a, b may be viscoelasticity due to collagen fibers moving within culture media, which is not included in our model in detail, and due to an increasing stiffness of the gel due to progressed polymerization when being placed in an incubator at 37°C for longer times. Finally, subtle aspects on the subcellular scale that are not included in our model may affect the time to reach the homeostatic state substantially because it is well known that this time differs considerably for different cell types (Eichinger et al. 2021).

**Fig. 7:**
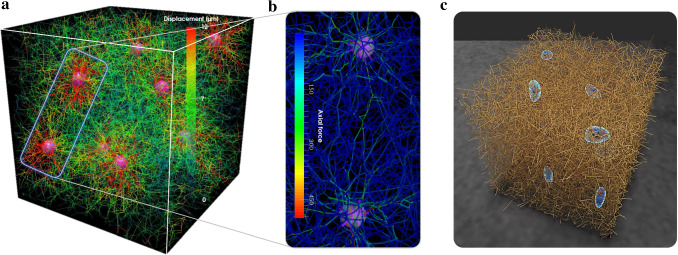
Cells mechanically interact with surrounding matrix fibers. **a** Cells attach to nearby fibers, contract, and thereby deform the matrix. The simulated, cell-mediated matrix displacements are in a realistic range when compared to experimental data (Notbohm et al. 2015; Malandrino et al. 2019). **b** Contracting cells can mechanically interact with other cells over a distance of several cell diameters via long-range mechanical signaling through matrix fibers, a phenomenon observed also in experiments (Ma et al. 2013; Shi et al. 2013; Baker et al. 2015; Kim et al. 2017; Mann et al. 2019). **c** Cells, visualized with reconstructed cell membrane around stress fibers, develop different shapes when pulling on the ECM

Figure 7a shows that the deformation of the matrix fibers around the cells in our simulations is on the order of $$10\ \text{$\mu$m}$$, which agrees well with experiments (Notbohm et al. 2015; Malandrino et al. 2019). Our simulation framework also reproduces the ability of cells to communicate via long-range mechanical interactions over several cell diameters (Fig. 7b), which has also been observed experimentally (Ma et al. 2013; Shi et al. 2013; Baker et al. 2015; Kim et al. 2017; Mann et al. 2019).

#### Variation of collagen concentration

**Fig. 8:**
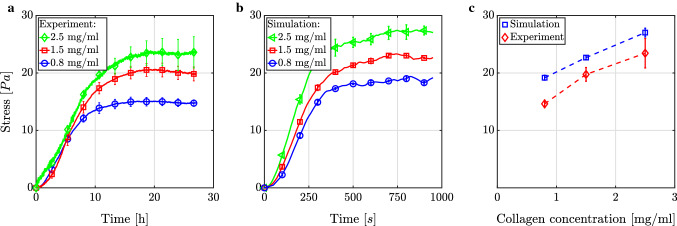
Mechanical homeostasis for a cell concentration of $0.5 \cdot 10^6\ \text {cells/ml}$ and different collagen concentrations in **a** experiments (Eichinger et al. 2020) and **b** our simulations. **c** In both cases, the relation between homeostatic first Piola–Kirchhoff stress (last data points were taken, respectively) and collagen concentration is approximately linear. All lines show the mean ± SEM of three identical experiments in **a** and **c** and of three simulations with different random network geometries in **b** and **c**

It is well known that interactions between cells and their environment crucially depend on the stiffness of the environment. This holds in particular for the proliferation, survival, migration, and differentiation of cells (Wang et al. 2012; Nguyen et al. 2018; Balcioglu et al. 2020). A simple way of testing the impact of stiffness on cellular behavior in tissue culture studies is to change the collagen concentration of the tested gels (Alcaraz et al. 2011; Miroshnikova et al. 2011; Hall et al. 2016; Joshi et al. 2018). As shown in Fig. 8a, tissue culture studies with a cell density of $0.5 \cdot 10^6\ \text {cells/ml}$ revealed that the cell-mediated first Piola–Kirchhoff stress increases in collagen gels with the collagen concentration (Delvoye et al. 1991; Eichinger et al. 2020). This behavior is both qualitatively and quantitatively reproduced well by our simulations as shown in Fig. 8b. Interestingly, both experiments and simulations exhibit a nearly linear relation (with a slope of $\sim 9/2$) between collagen concentration and the homeostatic stress (Fig. 8c). Moreover, the slope of the increase in stress up to the homeostatic stress was largely independent of the collagen concentration compared to the cell density in both the experiments and our simulations. We know from our simulations that an increased fiber density in cases of higher collagen concentrations in combination with a constant distance between integrin binding spots on fibers of 50 nm (López-García et al. 2010) leads to more cell–matrix links per cell over time (data not shown) even when considering only the mechanisms presented in Sect.2. If one assumes that a cell stresses fibers one by one up to a certain level, this process takes longer if more fibers are present and can explain the observed nearly linear relationship between homeostatic stress and collagen concentration as well as the similar initial slope for all three collagen concentrations.

### Residual matrix tension

**Fig. 9:**
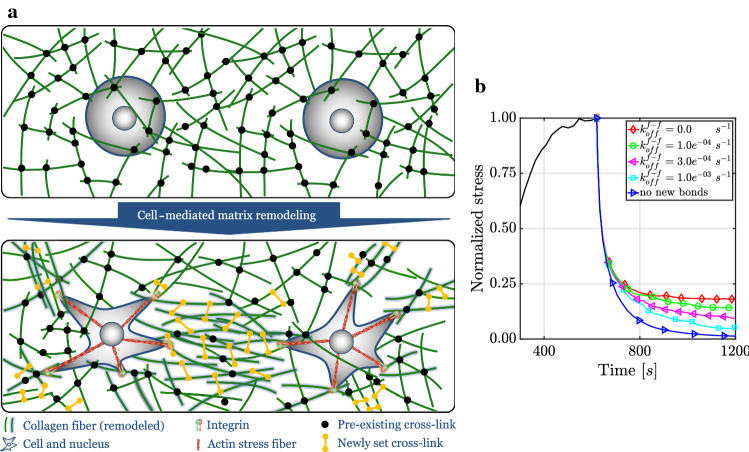
**a** Cells actively permanently remodel their surrounding by reorganizing the network and establishing new cross-links. This way, cell-mediated tension can be entrenched in the network. **b** When removing active cellular forces suddenly, the matrix tension quickly drops. However, if cells have entrenched their reorganization of the network structure by permanent (covalent) cross-links (i.e., with $k^{f-f}_{off}=0.0$), a residual tension persists in the network. By setting transient cross-links with a sufficiently low off-rate, the cells can ensure an RMT at least over the periods considered

**Fig. 11:**
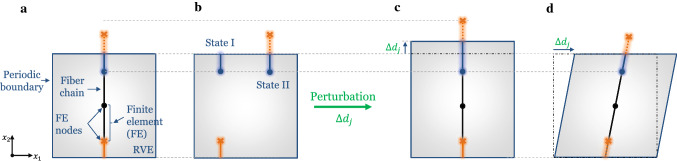
Illustration of periodic boundary conditions using the example of a single fiber in a network: **a** any fraction of an element sticking out of a periodic boundary must have a counterpart entering at the opposing side; **b** both element fractions together define what is physically present within the RVE (state I). To compute strains and stresses in both element fractions, it is convenient to use a fictitious state II (shifted rightward in the figure for illustration purposes only), which represents the part of the cut element within the simulated RVE and the part located in an adjacent domain periodically continuing the RVE; **c** application of fully periodic normal strain boundary condition in vertical direction; **d** application of fully periodic shear strain boundary condition in the drawing plane

Mechanical homeostasis in soft tissues is closely linked to growth (changes in mass) and remodeling (changes in microstructure) (Cyron and Humphrey 2017). In particular, a reorganization of the microstructure of tissues includes a change in the mechanical links between tissue fibers and of the constituent-specific natural (stress-free) configurations. Experimental studies have revealed that remodeling of collagen gels induced by cellular forces is time dependent and inelastic (Kim et al. 2017; Ban et al. 2018). Recent computational work suggested that the inelastic nature of cell-mediated remodeling is induced by force-dependent breaking of weak inter-fiber connections followed by the formation of bonds in new configurations leading to altered connections between tissue fibers (Nam et al. 2016; Kim et al. 2017; Ban et al. 2019; Cao et al. 2017) (Fig. 9a). This implies that after cell-mediated remodeling, a part of the matrix tension remains in the tissue even after the elimination of all active cellular forces (e.g., by disrupting the actomyosin apparatus via addition of cytochalasin D or by cell lysis). This remodeling is often referred to as residual matrix tension (RMT) (Marenzana et al. 2006; Simon et al. 2014).

To date, our quantitative understanding of how an altered state of the matrix is entrenched during remodeling and how RMT develops is limited. Even the exact kind of cross-linking which occurs when matrix tension is entrenched is unknown. An inelastic change of the stress-free configuration of the tissue could emerge from newly formed, transient bonds between collagen fibers (such as hydrogen bonds or van der Waals forces) as a result of fiber accumulation in the surroundings of contractile cells (Kim et al. 2017; Ban et al. 2018). However, RMT could also be entrenched by cells producing covalent cross-links via the actions of tissue transglutaminase, which can also form new bonds between deformed matrix fibers. The impact of these enzymes on matrix remodeling has been shown experimentally in free-floating collagen gels (Simon et al. 2014). To study RMT, we simulated the experimental protocol presented in Marenzana et al. (2006) and eliminated active cellular forces from the simulated system in the homeostatic state by dissolving all existing cell–ECM bonds at a certain time (by setting $k^{c-f}_{on} =0$, which led to a rapid dissolution of the remaining bonds). We then tracked tension over time in the RVE.

We first studied RMT in a purely covalently cross-linked network, implying that all existing bonds between fibers remained stable and no new bonds were formed during the simulation. After deactivating active cellular forces, we observed a (viscoelastic) decline of tension to zero in the RVE (Fig. 9b, bottom curve). This finding suggested that networks that lack the ability to form new, at least temporary stable, bonds cannot entrench a residual tension in the matrix, which was observed in the aforementioned experimental studies (Marenzana et al. 2006; Simon et al. 2014).

In a second step, transient linkers (which could, for example, be interpreted as un-bonded, freely floating collagen molecules or hydrogen bonds) were allowed to form between fiber to fiber binding spots with a certain on-rate $k^{f-f}_{on}$; they were able to be dissolved with a certain off-rate $k^{f-f}_{off}$. If two binding spots of two nearby fibers resided at some point in close proximity to each other, a new, initially tension-free bond was formed according to Eq. (2). We found that introduction of newly formed, transient bonds enables the entrenchment of matrix remodeling and thus some RMT (Fig. 9b, $k^{f-f}_{off}= 1.0e^{-04}\ s^{-1}$, $k^{f-f}_{off}= 3.0e^{-04}\ s^{-1}$, $k^{f-f}_{off}= 1.0e^{-03}\ s^{-1}$) at least for a prolonged period. The transient nature of the cross-links between the fibers resulted, however, in a slow decrease in RMT over time. This decrease happened faster the higher the off-rate $k^{f-f}_{off}$ (Fig. 9b). If $k^{f-f}_{off}$ was chosen above a certain threshold, we did not observe any RMT.

In a third study, we allowed covalent cross-linker molecules to form between two nearby collagen fibers when they were within a certain distance to each other and Eq. (2) was fulfilled. By setting $k^{f-f}_{off} = 0$, a newly set bond could not be dissolved and was therefore covalent (permanent). In this case, we observed a substantial RMT that apparently did not decrease over time (Fig. 9b, $k^{f-f}_{off}=0.0\ s^{-1}$).

It thus appears that both transient and covalent cross-links play roles in inelastic matrix remodeling. Our study suggests that RMT crucially depends on the ability of cells to entrench the deformation they impose on their neighborhood by covalent, permanent cross-links. Such a permanent entrenchment appears energetically favorable because it releases cells from the necessity of maintaining matrix tension over prolonged periods by active contractile forces, which consume considerable energy.

## Conclusion

To date, our understanding of the governing principles of mechanical homeostasis in soft tissues on short time spans especially on the scale of individual cells remains limited (Eichinger et al. 2021). To address some of the many open questions in this area, we developed a novel computational framework for modeling cell–ECM interactions in three-dimensional RVEs of soft tissues. Our computational framework generates random fiber networks whose geometric characteristics resemble those of actual collagen type I gels, that is, they exhibit a similar distribution of valency, free-fiber length, and orientation correlation (cosine) between adjacent fibers. These microstructural characteristics have been shown to be the primary determinants of the mechanical properties of fiber networks (Davoodi-Kermani et al. 2021). To model the mechanics of the collagen fibers in the network, our framework discretizes these fibers with geometrically exact nonlinear beam finite elements, which are shown in Sect. 3 to reproduce the elastic properties of collagen fiber networks. Our framework enables efficient parallel computing and can thus be used to simulate RVEs of tissues with realistic collagen concentrations and cell densities.

The physical interactions of cells with surrounding fibers through stress fibers in the cytoskeleton and transmembrane proteins (integrins) are modeled by contractile elastic springs whose binding and unbinding dynamics closely resemble the situation in focal adhesions. We used the non-trivial, experimentally determined relations of both cell density and collagen concentration to the homeostatic stress to show that the mechanisms accounted for in our computational framework are sufficient to capture these relationships. We also demonstrated how our framework can help to (quantitatively) examine the micromechanical foundations of inelastic cell-mediated matrix remodeling and RMT, which persists in the tissue even after active cellular forces have been removed.

Despite its advantages and broad experimental foundation, the proposed computational framework has some limitations that remain to be addressed. First, our model does not yet capture mass turnover, that is, the deposition and degradation of fibers, which are assumed to be crucial for mechanical homeostasis on long time scales (Humphrey and Rajagopal 2002; Ambrosi et al. 2011; Cyron et al. 2016; Braeu et al. 2017; Cyron and Humphrey 2017). Moreover, it models integrins but not associated proteins that also play a key role in the interactions between cells and surrounding matrix such as talin and vinculin (Ziegler et al. 2008; Grashoff et al. 2010; Carisey et al. 2013; Dumbauld et al. 2013; Das et al. 2014; Yao et al. 2014; Austen et al. 2015; Truong et al. 2015; Davidson et al. 2015; Zhu et al. 2016; Yao et al. 2016; Ringer et al. 2017). Also the model of cellular contractility is simplistic and should be endowed with additional biological details (Mogilner and Oster 2003; Murtada et al. 2010, 2012). Finally, we did not consider contact forces between fibers or between cells and fibers (assuming that cells and fibers mainly interact via integrins). While this reduces the computational cost substantially, a comprehensive incorporation of contact mechanics could also help to make our computational framework more realistic.

An important field of application for our computational framework will be *in silico* studies in which one can test step by step which additional features have to be incorporated in the framework to capture more and more phenomena observed *in vitro* and *in vivo*. In this way, it may contribute to uncovering the micromechanical foundations of mechanical homeostasis on the level of individual cells and fibers and help to understand how these microscopic processes lead to what we call mechanical homeostasis on the macroscale.

## Figures and Tables

**Fig. 10:**
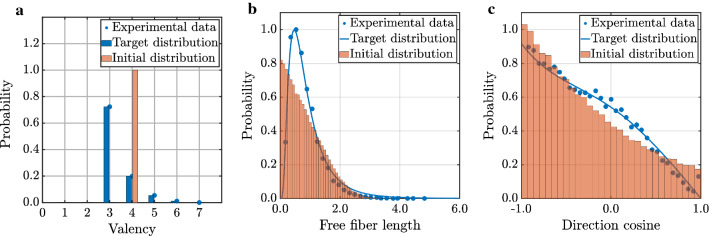
Random initial descriptor distributions in a network generated by Voronoi tessellation vs. target distributions fitted by Lindström et al. (2010) and Nan et al. (2018) to experimental data (collagen concentration 2.5 mg/ml). Simulated annealing alters the initial network until its descriptor distributions match the required target distributions

**Fig. 12:**
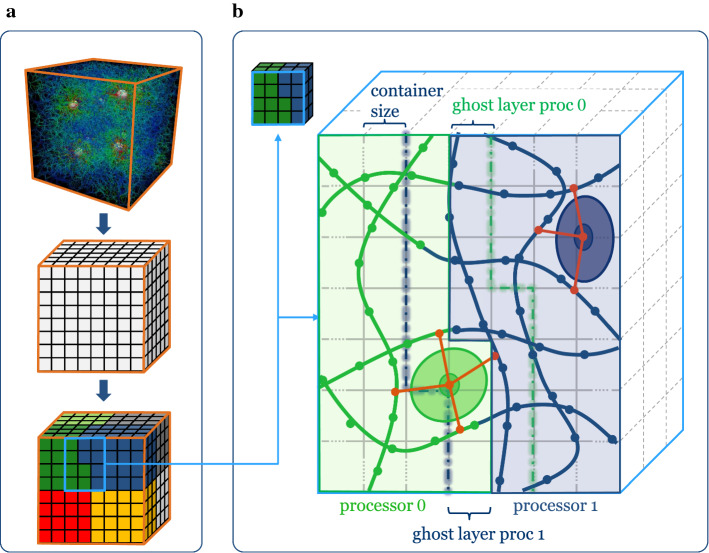
**a** Our computational domain (top) was divided into a large number of cubic containers (middle). Sets of numerous such containers (highlighted by different colors, bottom) were distributed to different processors. **b** All fibers discretized by beam finite elements as well as all cells were assigned to all containers with which they overlapped. Each processor was provided information not only about its own containers but also about a layer of ghost containers elements with which the elements in its own container may interact

## Data Availability

https://github.com/bionetgen/bionetgen.

## A1 Construction of random fiber networks by simulated annealing

In this appendix, we present the computational details of the algorithm we used for constructing network RVEs as an input for our simulations. Our algorithm closely follows the approach of Lindström et al. (2010), using the stochastic optimization method of simulated annealing for constructing random heterogeneous media introduced by Yeong and Torquato (1998). Thereby, one assumes that the geometry of a fiber network can be characterized by some descriptors ${\varvec{x}}_i$, with $i \in \{l, c\}$, in our case representing the fiber length and the direction cosine, respectively. These descriptors can be understood as random variables taking on specific values at certain nodes or fibers and characterize the network microstructure. The descriptors are assumed to follow some statistical distribution $P^{i}(x_i)$ across the different fibers and nodes. These distributions can be determined, for example, from confocal microscopy images of real networks (see also Fig. 10). According to Lindström et al. (2010), this yields for collagen type-I networks

7 $$\begin{aligned} P^l(l)&= \frac{1}{l\sigma \sqrt{2\pi }} \exp \left( -\frac{[\mu - \ln (l)]^2}{2\sigma ^2} \right) , \end{aligned}$$

where *l* denotes the fiber length normalized by $(N/V_{RVE})^{\frac{1}{3}}$, with $V_{RVE}$ being the volume of the RVE and *N* representing the total number of network nodes in it. The parameters $\sigma$ and $\mu$ denote a standard deviation and mean value that may vary from network to network. Typical parameters are given in Table 1. The cumulative probability distribution associated with $P^l(l)$ is given by

8 $$\begin{aligned} C^l(l)&= \frac{1}{2} + \frac{1}{2} {{\,\mathrm{erf}\,}}\left( \frac{\ln (x) - \mu }{\sqrt{2}\sigma } \right) \end{aligned}$$

and will be used in Eq. (13).

The distribution of the direction cosine $\beta$ of fibers adjacent to the same node has been described by Lindström et al. (2010) by a truncated power series

9 $$\begin{aligned} P^c(\beta )&= \sum _{k=1}^{3} b_k\left( 1-\beta \right) ^{2k-1}, \end{aligned}$$

with the associated cumulative distribution function

10 $$\begin{aligned} C^c(\beta )&= 1 + \sum _{k=1}^{3} -\frac{b_k}{2k} \left( 1-\beta \right) ^{2k-1}. \end{aligned}$$

Again, typical values for the parameters $b_k$ are given in Table 1. To describe the valency distribution of the networks, we relied on the data reported in Nan et al. (2018).

**Table 1:** Parameters for length, valency, and cosine distribution functions according to Lindström et al. (2010) and parameters used for simulated annealing process

Parameter	Description	Value [–]
$w_{l}$	Weight for free-fiber length distribution in Eq. (11)	1.0
$w_{c}$	Weight for direction cosine distribution in Eq. (11)	1.0
$\mu$	Mean in Eqs. (7) and (8)	$-0.3000$
$\sigma$	Standard deviation in Eqs. (7) and (8)	0.6008
$b_1$	Parameter for truncated power series in Eqs. (9) and (10)	0.6467
$b_2$	Parameter for truncated power series in Eqs. (9) and (10)	$-0.1267$
$b_3$	Parameter for truncated power series in Eqs. (9) and (10)	0.0200
$b_{l}$	Number of bins for free-fiber length distribution in Eq. (13)	1000
$b_{c}$	Number of bins for direction cosine distribution in Eq. (13)	1000
$T_{0}$	Initial temperature	0.05
−	Resulting average of nodal valency of constructed networks	3.3

Our target was to construct artificial random fiber networks as an input for our simulations whose descriptor distributions matched the ones defined above. To this end, we started from some random initial network. This network was then evolved in a number of discrete random steps according to the concept of simulated annealing (Kirkpatrick et al. 1983), until the descriptor distributions matched the desired target distributions.

To define the random initial configuration, we started by generating networks based on three-dimensional Voronoi tessellations (Rycroft 2009) with periodic boundary conditions applied in all directions. Subsequently, we randomly removed and added fibers until the valency distribution matched its target distribution. Only then we started the actual simulated annealing, where only fiber length and direction cosine distributions still had to be matched to their target distributions. The simulated annealing was performed following the concept introduced by Kirkpatrick et al. (1983). The idea is to iteratively select random nodes in the network and apply random displacements to them (Fig. 1b). In this way, the length of all fibers attached to the respective node and the angles between these fibers change. Importantly, only movements of nodes are accepted which do not lead to fiber lengths larger than one-third of the smallest edge length of the RVE to ensure that it stays *representative*. Note that a movement of a node does not affect its connectivity, which ensures that the initially created valency distribution remains unaffected during the whole simulated annealing.

For stochastic optimization according to the simulated annealing concept, it is helpful to define an objective (energy-type) function *E*

11 $$\begin{aligned} E&= w_l \cdot E^l + w_c \cdot E^c ,\end{aligned}$$

where the $E^l$ and $E^c$ become minimal if the length and direction cosine distribution exactly match their target distributions and where the $w_{i} > 0$ are weights that can be adapted to tune the importance of a specific distribution function. Having defined the objective function *E*, simulated annealing can be understood as a stochastic minimization of *E*. Once the minimum is found, $E^l$ and $E^c$ must be minimal and thus the length and direction cosine distributions match their target distributions. To perform a stochastic minimization of *E*, a Metropolis algorithm is applied during the simulated annealing. It consists of a sequence of random steps. For each of these steps, the associated change of *E* is computed, that is, $\Delta E$. Then, the step is actually performed only with a likelihood

12 $$\begin{aligned} p_{accept}(\Delta E)&= {\left\{ \begin{array}{ll} 1, &{} \Delta E \le 0 \\ \exp (-\frac{\Delta E}{T}), &{} \Delta E > 0, \end{array}\right. } \end{aligned}$$

where *T* denotes a temperature-like parameter (having units of energy). In our simulated annealing, we slowly decreased *T* as the number random steps increased, using at the annealing step *k* the value $T = 0.95^k \cdot T_0$ (according to Nan et al. (2018)). We chose $T_0$ such that the probability for accepting a random step with $\Delta E > 0$ was approximately 0.5 in the beginning. In practice, the simulated annealing was stopped if either the total energy of the system was below a predefined threshold or a maximal number of iterations were reached.

### Remark A1

It is worth noting that for constructing RVEs with different collagen concentrations, we assumed the same target distributions for the valency, direction cosine and normalized fiber length. Only the normalization factor of the fiber length was changed. Moreover, an increased collagen concentration automatically also implies a higher number *N* of network nodes in the RVE.

### Remark A2

While there exists a variety of simple and obvious choices for the $E^i$ in Eq. (11), these mostly suffer from a computational cost on the order of ${\mathcal {O}}(n_i)$ with $n_i$ the number of instances of a descriptor. This makes the generation of large random networks practically infeasible. To overcome this problem, we adopted the idea of Lindström et al. (2010) to use a binning algorithm and define the $E^i$ as Cramer–von Mises test statistics, which reduces the computational cost to the order of ${\mathcal {O}}(b)$ with *b* the number of bins. To this end, we divided the range of ${\varvec{x_i}}$ in $b_i$ disjoint intervals (bins) and assigned each instance of a descriptor at a fiber or node of a random network to its associated bin. The resulting histogram is a discrete approximation of $P^i(x_i)$. The center of the *j-*th bin is denoted by $x_{ij}$. The number of instances of descriptor $x_i$ assigned to the *j*-th bin is $m_{ij}$. The Cramer–von Mises test statistics can then be computed as

13 $$\begin{aligned} \begin{aligned} E^i = \frac{1}{n_i^2}\sum _{j=1}^{b_i} m_{ij} \left[ \frac{1}{6}(m_{ij}+1)(6S_{ij}+2m_{ij}+1)+S_{ij}^2 \right], \end{aligned} \end{aligned}$$

with $S_{ij} = M_{i(j-1)} - nC^i(x_{ij}) - \frac{1}{2}$, $M_{ij} = \sum _{k=1}^{j}m_{ik}$, and $C^i$ the cumulative distribution of $x_i$.

## A2 Boundary conditions

Here, we summarize how we applied fully periodic boundary conditions to our simulation domains. Let these domains be cuboids with edge length $L_i$ in the *i*-th coordinate direction. In a fully periodic network, the part of a fiber sticking out across one periodic boundary must have a counterpart entering the RVE at the opposing side (Fig. 11a). One can interpret the element part sticking out of the domain at one boundary and the element part entering the RVE at the opposing boundary also as a fictitious single element (Fig. 11b, state II) cut into two parts (Fig. 11b, state I). Thereby, state I as delineated in Fig. 11b can be used to evaluate interactions with other fibers or cells, and state II for evaluating strains and stresses on element level. If the element is cutting through a boundary in the *i-﻿*th coordinate direction, the *﻿i*-th coordinate of the nodal positions in states I and II is shifted by $L_i$ relative to each other. Importantly, only the translational degrees of freedom of the beam finite element nodes are affected by the periodic boundaries, rotational degrees of freedom remain unaffected.

It is a major challenge to impose periodic Dirichlet boundary conditions on fiber networks in a manner that is fully periodic. Most of the literature (Stein et al. 2010; Lee et al. 2014; Abhilash et al. 2014; Liang et al. 2016; Humphries et al. 2018; Burkel et al. 2018; Ban et al. 2018, 2019) bypasses this difficulty by fixing nodes on or close to the periodic boundary in a manner that unfortunately cannot ensure periodicity in a rigorous manner. To overcome this deficiency, we used the following approach. Dirichlet boundary conditions on RVEs can be represented by normal or shear strains. These strains can be converted into a relative displacement of opposing periodic boundaries by components $\Delta d_j$ in the *j*-th coordinate direction. We accounted for this displacement by stretching (Fig. 11c) or shearing (Fig. 11d) the RVE as a whole. The nodal positions in states I and II were then no longer converted into each other in the above described simple manner, that is, by a relative shift by $L_i$ in the *i*-the direction. Rather, all coordinates of the nodal positions were additionally shifted relative to each other by the components $\Delta d_j$. Note that this approach can account for complex multi-axial loading by applying the described procedure at all periodic boundaries. Moreover, this approach can account for large strains.

## A3 Search algorithm and parallel computing

Here, we describe how we ensured efficient parallel computing for the presented modeling framework in our in-house finite element solver BACI 2021. Parallelization of the finite element discretization of the fibers can be handled with standard libraries such as the Trilinos libraries that form the basis of our in-house code. Therefore, we focus on the parallelization of cell–fiber interactions and chemical bonds between fibers; both require search algorithms to identify cell–fiber or fiber pairs that may interact at a certain point in time. We implemented a search algorithm based on a geometrical decomposition of the computational domain (RVE) in uniform cubic containers. For simplicity, these were aligned with the axes of our coordinate system (Fig. 12). Cells and finite beam elements are assigned to all containers with which they overlap. We chose the minimal size of the containers such that all possible interaction partners were certainly located within one layer of neighboring containers. Hence, evaluating the possible interactions of a single cell or beam finite element simply required searching within one layer of containers around the containers to which the cell or element was assigned.

The content of the containers had to be updated over time as cells and matrix fibers moved during our simulations. Depending on the time step size of our simulations, container size and effective physical interaction distance, it was feasible to update our containers only every *n-*th time step.

The potentially large domain considered in our simulations typically required a distribution of the above described containers on several processors. To this end, each processor was assigned a set of containers forming a connected subdomain. In addition to these containers, each processor was also provided full information about one layer of so-called ghost containers surrounding its specific subdomain (Fig. 12b). The computational cost of sharing the information about ghost containers was negligible compared to the overall computational cost of our simulations.

To enable an effective search algorithm based on a rectangular Cartesian domain, we used a coordinate transformation to the undeformed domain in case boundary conditions imposing a deformation of the computational domain.

It is worth mentioning that in our parallelization framework, no data (except some uncritical parameters such as the current time step) need to be stored fully redundantly on all processors, which would drastically limit the problem sizes.

## A4 Simulation parameters

**Table 2:** List of parameters and default values of computational model

Parameter	Description	Value	References
$a_1$	Integrin catch–slip bond parameter	2.2	To fit data of Kong et al. (2009)
$b_1$	Integrin catch–slip bond parameter	29.9	To fit data of Kong et al. (2009)
$c_1$	Integrin catchslip bond parameter	8.4	To fit data of Kong et al. (2009)
$a_2$	Integrin catch–slip bond parameter	1.2	To fit data of Kong et al. (2009)
$b_2$	Integrin catch–slip bond parameter	16.2	To fit data of Kong et al. (2009)
$c_3$	Integrin catch–slip bond parameter	37.8	To fit data of Kong et al. (2009)
*R*	Cell radius	12 μm	Typical value
$\Delta R$	Linking range around cell	±3 μm	–
$D_{f}$	Diameter of collagen fibers	180 nm	Van Der Rijt et al. (2006)
$E_{f}$	Young’s modulus of collagen fibers	1.1 MPa	Jansen et al. (2018)
$${\dot{c}}$$	Contraction rate of stress fibers	$$0.1 \frac{{\hbox{$\mu$ m}}}{\hbox{s}}$$	Choquet et al. (1997); Moore et al. (2010)
$k_{B}T$	Thermal energy	$4.28 \cdot 10^{-3}\ \rm{aJ}$	at 37$^\circ \,\hbox {C}$
$L_i$	RVE edge length in *i*-th coordinate direction	$245 \mu m$	–
$k^{f-f}_{on}$	Chemical association rate for fiber linker	$0.0001 s^{-1}$	–
$k^{f-f}_{off}$	Chemical dissociation rate for fiber linker	$0.0001 \rm{s}^{-1}$	–
$\Delta x$	Bell parameter	0.5 nm	–
$N_{FA,max}$	Maximal number of focal adhesions per cell	65	Kim and Wirtz (2013); Horzum et al. (2014); Mason et al. (2019)
$N_{i,FA,max}$	Maximal number of integrins per focal adhesion	1000	Wiseman (2004); Elosegui-Artola et al. (2014)
$N_{i,ic,max}$	Maximal number of integrins per cluster	20	Changede et al. (2015); Cheng et al. (2020)
$k^{c-f}_{on}$	Chemical association rate for integrin	$0.1\ s^{-1}$	slightly modified Zhu et al. (2016)
$d^{i-f}$	Distance between binding spots for integrinfiber links	50 nm	López-García et al. (2010)
